# Factors accounting for limited sexual reproduction in a long-lived unisexual plant species

**DOI:** 10.3389/fpls.2025.1456877

**Published:** 2025-02-11

**Authors:** Irene Bisang, Flavien Collart, Alain Vanderpoorten, Lars Hedenäs

**Affiliations:** ^1^ Department of Botany, Swedish Museum of Natural History, Stockholm, Sweden; ^2^ Department of Ecology and Evolution, University of Lausanne, Lausanne, Switzerland; ^3^ InBioS, Institute of Botany, University of Liège, Liège, Belgium

**Keywords:** *Abietinella abietina*, bioclimatic gradient, bryophytes, niche modelling, niche similarity, phenotypic sex ratio, sexual reproduction, topography

## Abstract

**Introduction:**

Plant dispersal directly depends on reproduction success, and hence, on sexual systems. In bryophytes, wherein fertilization involves a continuous film of water between male and female sexual organs, reproduction in unisexual species involves the sympatric distribution of male and female sex-expressing individuals. Here, we determine whether these conditions are controlled by the environment. In particular, we test the hypotheses that (i) sex-expressing males and females exhibit different ecological niches and (ii) environmental variation drives sex expression, sporophyte formation, and hence, dispersal capacities.

**Methods:**

We scored 1,080 specimens of the unisexual moss *Abietinella abietina* across Sweden as non-sex expressing, expressing female or male, or sporophytic. We tested whether reproductive stages were related to latitude. Topography and climatic conditions at 1-km resolution were employed to measure niche overlap between (i) sex-expressing and non-expressing and (ii) male and female specimens. We finally modelled sex expression and sporophyte production depending on these topo-climatic predictors.

**Results:**

Among the 63% of reproductive samples across the entire latitudinal gradient, females outnumbered males by a factor 5.6, and 8% of the female samples bore sporophytes. Although the distribution of the sexes was not explained by topo-climatic variables, the probability of sex-expressing samples being male increased with latitude. It resulted in a higher regional sex ratio in the North than in southern regions. Successful sexual reproduction, in terms of sporophyte occurrence, was confined to central Sweden. It was predicted by intermediate to increasing precipitation seasonality and intermediate temperature values.

**Discussion:**

Despite a high level of sex-expression, and no significant differences of niche preference between males and females, sporophyte occurrences were rare. Our results suggest that sporophyte formation was determined by mate availability and macro-climatic conditions, the latter possibly affecting fertilization success. We further infer that environmental conditions at the pre-zygotic stage have lower than expected effects on the overall distribution of this moss. Modelling environmental data at higher resolution, smaller scale and expanding geographic coverage to include more sporophyte occurrences, and comparing genetic diversity in sporophytic with non-sporophytic populations, are future lines of this research.

## Introduction

**Figure 1:**
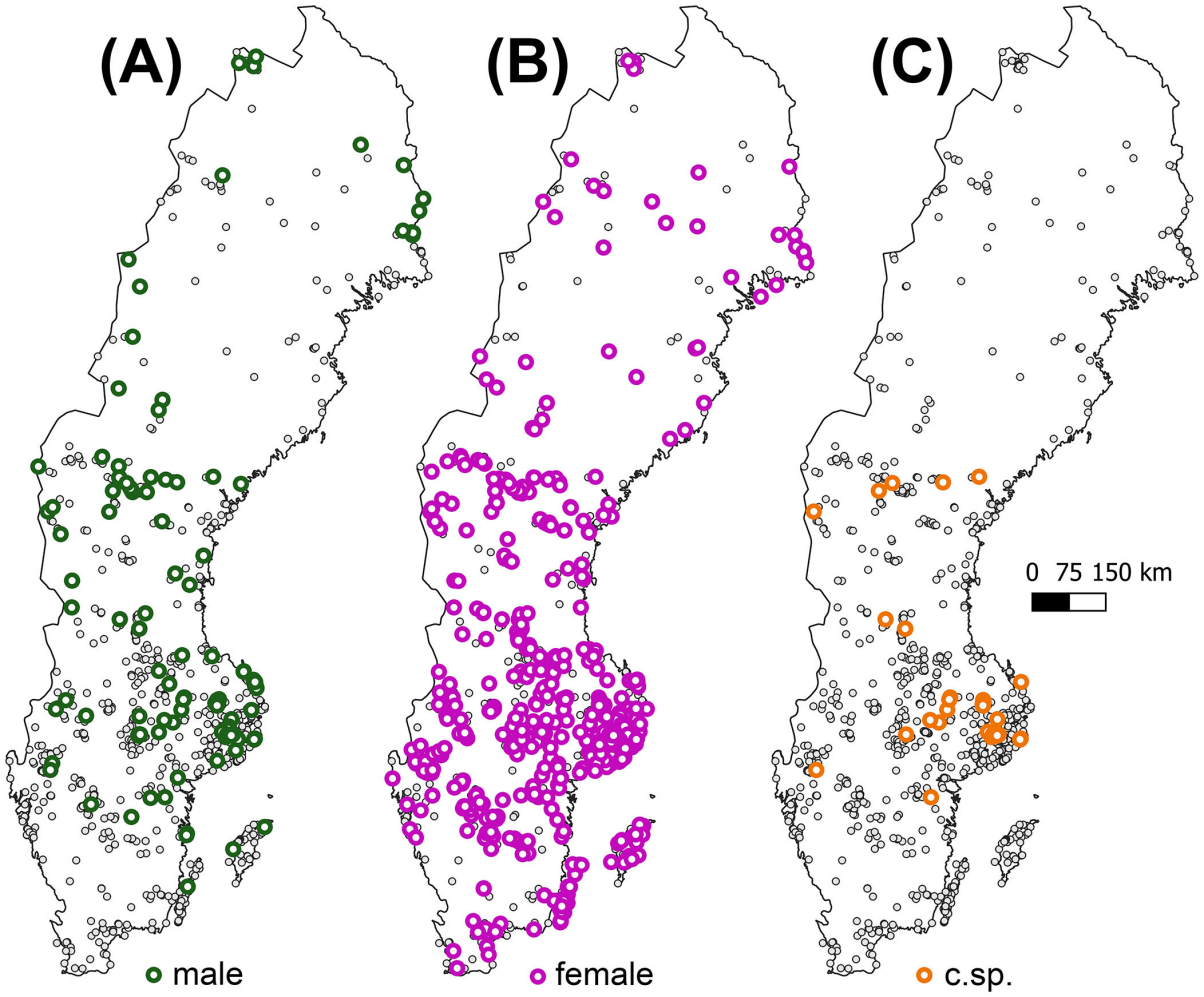
The distribution of **(A)** male expressing, **(B)** female expressing, and **(C)** sporophytic samples of *Abietinella abietina* across Sweden (n = 1,080). Localities of non-reproductive samples are displayed by small grey dots. For locality data, see **Supplementary Table S1**.

Dispersal is a key process determining species distributions and geographic ranges, community structure, population dynamics and transmission of genetic variation (e.g., Eckert, 2002; Munoz et al., 2004: **Figure 1**; Lewis et al., 2014; Barrett, 2015; Beckman et al., 2018). In plants, dispersal and reproduction are tightly associated as the production of diaspores results from reproduction, either sexual, asexual or both (Grossenbacher et al., 2015; Löbel et al., 2018). The reproductive mode, and the balance between different reproductive modes, depend on species-inherent traits, such as genotype or age, biotic factors, e.g. competition, herbivory or pollinator attributes (Dorken and Eckert, 2001; López et al., 2001; Zhang and Zhang, 2007; Silvertown, 2008; Buchanan, 2015) and these are affected by environmental conditions (e.g., Tomita and Masuzawa, 2010; Yang and Kim, 2016). Dispersal abilities are influenced by the mode of reproduction through traits of the dispersal units produced by sexual vs asexual reproduction, and their establishment prospects, which both, in turn, also interact with conditions in the environment (Barrett, 2015; Beckman et al., 2018; Patiño and Vanderpoorten, 2018). In the case of sexual reproduction, both pre- and post-zygotic processes contribute to the dispersal of species and their genetic variation through active or passive dispersal of pollen and gametes (e.g., Cronberg, 2012; Aguilée et al., 2016) or seeds and spores (e.g., Schupp et al., 2010; Zanatta et al., 2016).

Bryophytes form a sister clade to all vascular land plants (tracheophytes), comprising three lineages, mosses, liverworts and hornworts. They are the only land plants with a life cycle dominated by the free-living multicellular haploid gametophyte (rather than the diploid sporophyte), and with sex manifested in the haploid gametophyte. This has a fundamental bearing for reproduction and dispersal processes, that differ from other embryophyte lineages (Haig, 2016; Fichant et al., 2023). The two major reproductive modes of bryophytes, asexual and sexual, play partly complementary roles for species’ population maintenance, sex ratios and dispersal (Lönnell et al., 2014; Laenen et al., 2015; Patiño and Vanderpoorten, 2018). Asexual reproduction is common and manifold, such as by multiplication of ramets, fragmentation or specialized vegetative diaspores (Laaka-Lindberg et al., 2003; Frey and Kürschner, 2011; van Zuijlen et al., 2023). It is thought to contribute mainly to short-distance dispersal and local population persistence. Sexual reproduction results in the production of spores, which, with an average size of 20 µm, are easily wind-borne and contribute to long-distance dispersal, shaping species’ ranges and community structures at the landscape level (Patiño and Vanderpoorten, 2018). However, successful sexual reproduction and the potential for long-range dispersal, rely on that sporophytes and spores are produced, and this is conditioned by a series of factors.

In many instances, gametophytic ramets (i.e., a vegetative unit of a clone capable of independent growth) do not form gametangia (i.e., structures bearing gametes). Plants that lack visible sexual organs, while retaining the potential for sexual reproduction, are in this context termed “non-expressing”. Sex expression is, at least in part, determined by environmental conditions (Boquete et al., 2023; Bisang et al., 2020), while in some species, sexual organs remain completely unidentified (Bisang and Hedenäs, 2005; Haig, 2016; de Jong et al., 2018). Furthermore, bryophyte sperm dispersal distances are with few exceptions limited to the scale of decimeters up to one meter (Bisang et al., 2004; Pressel and Duckett, 2019). Water is required for the sperm to travel to the sessile egg cell, although a fraction of sperm cells is tolerant to desiccation for extended periods (Shortlidge et al., 2012; Haig, 2016). Successful fertilization thus also strongly depends on environmental conditions (Sundberg, 2002; Hedenäs and Bisang, 2019). Around 60 to 70% of bryophyte species possess unisexual gametophytes (dioicous; compared to 4-6% dioecious sporophytes in seed plants; Wyatt, 1985; Renner, 2014; Laenen et al., 2016). Offspring sex determination occurs at meiosis in the sporophyte, when the heteromorphic U and V chromosomes segregate rather than at syngamy (Bachtrog et al., 2011; Renner et al., 2017). Although this process should lead to balanced progeny sex ratios, sex ratios in adult bryophyte populations are typically biased, usually with female dominance (Bisang and Hedenäs, 2005; Bisang et al., 2014). Species-specific life histories, migration history, ancestry or stochastic events, in relation to varying environmental conditions, may account for establishing and maintaining the common population sex ratio biases (Field et al., 2013; Yakimowski and Barrett, 2014; Bisang et al., 2023; Boquete et al., 2023). Effects of these factors on haploid sex ratios have rarely been addressed, and accordingly, knowledge of the consequences of sex ratio bias on species’ dispersal capabilities in bryophytes is still limited. Moreover, whether the widespread female population sex bias in bryophytes is due to true male rarity, or differential sex expression levels in males and females, remains poorly explored (Bisang et al., 2017; Nieto-Lugilde et al., 2018; Ekwealor et al., 2022). Genetic markers to identify the sex in non-expressing bryophytes were only recently developed (Korpelainen et al., 2008).

In unisexual species, fertilization may be further hampered by that individuals of the opposite sex need to be in close sympatry given the short sperm travel distances, by the fact that male and females differ in their morphology, physiology and possibly in niche preferences, and therefore tend to be spatially segregated (e.g., Stark et al., 2009; Bisang et al., 2015; Slate et al., 2017). Taken together, this imposes a series of severe constraints for fertilization to take place, including species’ level of sex-expression, spermatozoid moving distance, sex-ratio biases, spatial arrangement of the sexes, and whether spores of both sexes establish at the new place.

In this study, we focus on whether pre-zygotic reproductive traits (sex expression) in the unisexual moss *Abietinella abietina* (Hedw.) M. Fleisch. and environmental factors affect the success of reproduction along a bioclimatic gradient from southern to northern Sweden. *Abietinella abietina* is widely distributed across the Holarctic, inhabiting base-rich substrates in relatively open terrestrial environments. Successful sexual reproduction, indicated by the occurrence of sporophytes, is globally uncommon and regionally rare or absent (Lieske, 2010; Buck, 2014), but occurs abundantly and regularly in limited areas in Scandinavia (Hedenäs et al., 2014), making the species a suitable candidate to study reproductive performance in relation to environmental variation. We assessed the numbers and distribution of the reproductive stages sex-expressing vs non-expressing; sporophytic vs non-sporophytic females and phenotypic males vs phenotypic females of *A. abietina* in Sweden. Specifically, we asked 1) What is the proportion of samples with sexual organs (sex-expressing samples)? How many female samples carry sporophytes? How many of the sex-expressing samples are males or females? 2) Is the distribution of the reproductive stages (sex-expressing vs non-expressing; phenotypic males vs phenotypic females; sporophytic) spatially even across the study area or does it follow a latitudinal gradient? 3) If reproductive stages are not evenly distributed, can their different distributions be explained by environmental (climatic or topographic) conditions? Based on extensive field observations, surveys of natural history collections, and previous studies on this species (Hedenäs, 2000; Bisang et al., 2004; unpubl. data LH, IB), we expect that 1) there is a considerable proportion of non-expressing samples, females are dominant among the reproductive samples, and sporophytes are overall rare, and 2) the distribution of male and female sex-expressing and sporophytic samples are unevenly distributed across the study area. Finally, we hypothesize that 3) the occurrence of sex expression and sporophytes, and possibly of phenotypic females and males, is related to certain environmental conditions.

## Materials and methods

### Study area

The study area extends over most of Sweden, from southern Scania to the Lake Torneträsk area and covers the Swedish occurrences of the study species from 55.47°N to 68.52° N (**Supplementary Table S1**). It covers a climatic gradient from warm-temperate (nemoral), humid with warm summers climate zone in the south to the dominating cold-temperate (snow), humid with cool summers, climate zone in the north. The cold-temperate climate zone includes a polar tundra climate in the mountain range above the tree line (following Köppen-Geiger climate classification; Dierssen, 1996; Kottek et al., 2006). This includes an altitudinal range from sea level to almost 1200 m a.s.l. The degree of continentality (sub-oceanic to sub-continental) varies between coastal, mountain and inland areas (Dierssen, 1996).

### Study species

*Abietinella abietina* is a long-lived unisexual pleurocarpous moss widely distributed across the Holarctic (and reported from South Africa; Hedenäs et al., 2014). It grows as loose wefts on base-rich to calcareous ground, boulders, rocks, or occasionally at tree bases. It occurs in both exposed and shaded habitats, often at sites that regularly fall dry during summer, across whole Sweden. It is common in the south and parts of the mountain range in the north, and rarer in the northern lowlands. Sexual organs, gametangia, are surrounded by specialized leaves and form reduced branches, i.e., male perigonia or female perichaetia. Previous observations in the field and of natural history collections showed that sporophyte formation varies in time and geographically: Among almost 700 A*. abietinella*-samples collected until 1935, 33 specimens from 22 localities carry sporophytes. No samples with sporophytes were collected between 1936 and 2010 (N = 256). Since 2010, sporophytes were noted in 15 out of 132 samples from geographically restricted localities (https://artportalen.se/ViewSighting/SearchSighting; accessed 5 November 2023; unpubl. data LH, IB).

### Trait data collection and analyses

We scored the reproductive stage of the individual samples as sporophytic (realized sexual reproduction), with female pre-zygotic sexual branches, perichaetia (phenotypically female samples), with male pre-zygotic sexual branches, perigonia (phenotypically male samples), and non-sex expressing samples. We used herbarium collections from Sweden stored at the Swedish Museum of Natural History (S) collected until 1 April 2022. From the entire holdings of the study species at S up to this date, we discarded duplicate specimens, multiple collections from the same locality and specimens containing only a few shoots, but did not consider other features of the specimens (e.g., specimen size or label information other than locality data). We studied each sample under the dissecting scope until we noted reproductive structures, or up to a maximum of 15 minutes. We recorded latitude and longitude from label information if present, or picked the coordinates from the map of Sweden ‘Sveriges länskarta’ (https://ext-geoportal.lansstyrelsen.se/standard/?appid=7b933d2ea9084c4dab4bfe38dd87f7ec; accessed 11 October 2023). We mapped the samples using QGIS 3.28.4-Firenze (https://qgis.org/en/site/; accessed 11 October 2023).

This resulted in N = 1,080 studied specimens covering the bioclimatic gradient described above (**Supplementary Table S1**). Each specimen, typically up to c. 0.5 dm^2^, represents an individual patch of the species, hereafter termed ‘sample’. In its natural environment, *A. abietina*-patch size is variable, from small patches to a typical area of 1–2m^2^ and sometimes up to of several m^2^. Sexual branches and sporophytes persist for at least two to three reproductive cycles in the field and remain in dried specimens, once collected. Pre-zygotic reproductive organs are not easily detected in the field, and it is unlikely that their occurrence affected the collection behavior of bryologists. Sporophytes, except the youngest stages, are recognizable during field collection, and sporophytic plants might be collected preferably in species like *A. abietina* with rare sexual reproduction. However, there is no obvious reason that a potential collection bias should depend on geographical location. We thus consider the large number of samples across the study region a random representation of the species’ occurrences and appropriate to assess its reproductive patterns in the study region and to model niches of different reproductive stages based on presence data (niche analyses) or presence/absence data for the GLM and Random Forest analyses, respectively. For further discussion of the use of natural history collections for trait analyses, see Bisang et al. (2023; p 32, and references).

For visualization purposes, we classified the samples as to the three regions Southern (N = 227; c. 80,000 km^2^), Central (740; c. 175,000 km^2^) and Northern Sweden (113; c. 155,000 km^2^; **Supplementary Table S1**). The Central region is delimited by the occurrence of sporophytes (**Figure 1**; **Supplementary Figure S1**). The different sample sizes approximately reflect the frequency of the species in the regions, although northern Sweden may be underexplored.

We counted samples of each reproductive stage and calculated proportions of sex-expressing samples, and of female samples with sporophytes (**Supplementary Table S1**; **Figure 1**). We computed phenotypic (or functional) sex ratios as the number of male-expressing samples divided by the total number of sex expressing samples (Bisang et al., 2020; **Figure 1**); both across the entire study region, and for each sub-region (North, Central, South; **Supplementary Figure S1**) separately. Using this equation [M/(M+F)], sex ratio values <0.5 denote female-biased, values > 0.5 designate male-biased sex ratios, respectively. We considered each sample as one observational unit, which implies that we do not present variation metrics. We assigned samples containing both plants with perigonia and plants with perichaetia to both the male and female reproductive stages. Thus, total sample numbers for the reproductive stages ‘female’ and ‘male’ were higher than the actual numbers of sex-expressing samples. We calculated sex ratios excluding sporophytic samples to account for a potential over-estimation of the rarer sex in case sporophyte-bearing occurrences were preferably collected by experts (Bisang et al., 2014, 2023). We tested whether sex ratios differed from an expected unbiased sex ratio (0.5) with Pearson’s χ^2^ tests. We first inspected the distribution of the reproductive stages visually to explore potential spatial patterns. We then tested whether the probability of a sample to express sex and among the sex-expressing samples, to be male or female, with a generalized linear model (GLM) with a binomial distribution and a log-link function. In these analyses, we also excluded sporophytic samples. We modelled the probabilities both of being male and of being female. Because 13 non-sporophytic samples contained male and female sex organs (i.e., being female [male] is not equal to being non-male [non-female]), the exact estimates in the model results differ slightly, and we present the model for male expression probability here. To visualize the latitudinal gradient in phenotypic males, we compared sex ratios between the three regions outlined above with Pearson’s χ^2^ tests (North, Central, South; **Supplementary Figure S1**). Finally, we tested if the probability of sporophyte occurrences among female samples was related to latitude with a GLM with a binomial distribution and a log-link function, including polynomial predictors to account for the non-linear relationship. We performed the analyses using Statistica v.13.5 (TIBCO_Software_Inc., 2018).

### Climate and topographic data collection

To consider the temporal climate variability across the sampling period, we first generated monthly precipitation and minimum and maximum temperature values at 30-arc-second resolution (c. 1 km) for Sweden for each year between 1850 and 2015, using the CHELSA CMIP6 module (Karger et al., 2017; https://gitlabext.wsl.ch/karger/chelsa_cmip6) in Python v 3.8 (Van Rossum and Drake, 2009). We employed two historical global circulation models (GCMs: MPI, Mauritsen et al., 2019 and UKESM, Sellar et al., 2019) to perform the climate downscaling. Monthly climate parameters were then averaged across 20 years between 1850 and 2015 (1850-1870; 1871-1890;….; 1991-2015) and the 19 bioclimatic variables were generated (**Supplementary Table S2**). The 19 bioclimatic variables were designed to provide biologically meaningful variables of precipitation and temperature (Booth, 2022). We then extracted the 19 bioclimatic values for each sampling locality according to the sampling date.

For topography, we downloaded 12 topography metrics at 30-arc-seconds from Amatulli et al. (2018). These topographic variables capture various dimensions of relief, such as elevation, slope orientation (aspect), roughness, curvature and land position indices (detailed list in **Supplementary Table S2**). Given the accuracy of the locality data on the labels, we used spatial metrics at a resolution of 30-arc-seconds (c. 1 km) rather than finer resolution levels, to keep a potential geographic bias in the analyses low.

### Modelling niche overlap

To summarize the climatic and topographic spaces, we performed three Principal Component Analyses (PCA) using all the climatic values from the different time periods available across Sweden for both (i) MPI and (ii) UKESM circulation models, and (iii) all topographic values in Sweden. For the climatic (MPI or UKESM) and topographic spaces, the two first axes were kept, explaining 81%, 80% and 51%, respectively, of the total variance. We afterwards computed a climate niche and topographic niche overlap between males (144) and females (595) using Schoener’s D and Hellinger’s I metrics. We tested the significance of these overlaps using a niche similarity test (Broennimann et al., 2012) based on a null distribution of 1000 replicates and using the ecospat package (Broennimann et al., 2023) in R v4.2.2 (R-Core-Team, 2022). We used the same approach to calculate and test niche overlaps between sex-expressing (402) and non-expressing (678) samples.

### Modelling the response of sex expression and successful sexual reproduction by climatic and topographic factors

To determine the best climatic and topographic predictors of sex expression and of successful sexual reproduction, we performed a variable selection in two steps following Adde et al. (2023). We compared samples with male and/or female and without reproductive organs (N=676 [2 samples without topo-climate data, due to closeness to waterbodies] and N=401 [1 without topo-climate data]) and female samples with sporophytes present and without sporophytes (N=41 [7 sample localities without topo-climate data] and N=547) (**Supplementary Table S1**). To avoid multicollinearity issues, we first performed univariate Generalized Linear Models (GLM) with linear and quadratic terms and ranked each predictor based on their *p*-values (Adde et al., 2023). We kept the best predictor and removed all the other predictors that have a Pearson correlation > 0.7 with it (Dormann et al., 2013). We then selected the second best, removed the correlated predictors and continued this selection until no correlated predictors remained. With this subset of predictors, we then modeled the occurrence of sex expression and sporophytes using two different approaches, which allowed to select the best model structure: a GLM with elastic-net regularization (Zou and Hastie, 2005), allowing linear and quadratic terms, and a guided Regularized Random Forest (RRF; Deng and Runger, 2013) via glmnet and RRF packages (Friedman et al., 2010; Deng and Runger, 2013; Tay et al., 2023), following Adde et al. (2023). RRF provide high quality feature selection on top of the advantages of Random Forest models, such as high predictive accuracy and the ability to model complex interactions and non-linear relations (Izquierdo-Verdiguier and Zurita-Milla, 2020). For the successful sexual reproduction models, as the prevalence was relatively low (0.07), we gave weights for each sample to set prevalence at 0.5. We evaluated the fit of the models with the Area Under the Curve (AUC) and the maximum value of the True Skill Statistic (maxTSS) with the dismo and ecospat packages, respectively (Broennimann et al., 2023; Hijmans et al., 2023). We then plotted the response curve for each predictor and model by predicting the probability of sex expression and of sporophyte occurrence between the minimal and maximal value of the predictor, while keeping the remaining predictors constant using their median values (Elith et al., 2005). These models were generated twice by changing the global circulation model used for the climate data.

## Results

Among the total of 1,080 scored samples, 63% (678) carried sexual organs, i.e., were sex-expressing. Forty-eight samples bore sporophytes, which corresponded to 8% of female sex-expressing samples and indicated successful sexual reproduction (**Figure 1**; question 1). Excluding the samples with sporophytes, 61% (630 out of 1,032) samples expressed sex.

Among the non-sporophytic but sex-expressing samples, females were much more numerous and appeared more evenly distributed than males (547 [85%] vs 96 [15%]; 13 samples with both sexes) at the sampled localities across the entire country (**Figure 1B**; question 1). It resulted in a strongly female-skewed overall sex ratio of 0.15 (χ^2^ = 316.33; P<0.001). The regional sex ratios were also female-biased and increased from South (0.07; χ^2^ = 96.49; P<001), Central (0.15; χ^2^ = 223.81; P<0.001) to North Sweden (0.32; χ^2^ = 8.14; P=0.004) (**Supplementary Figure S1**; question 2). This is an effect of a higher probability of sex-expressing samples being male than females with increasing latitude (**Table 1A**). Despite males being absent from the northern inland and coastal areas, their relative proportion among sex-expressing samples is highest in the North. In south Sweden, male samples are about 20-fold rarer relative to females and absent in the southwest (**Figure 1A**). The probability to express sex was not associated with latitude (**Table 1B**, question 2). Successful sexual reproduction (48 sporophyte occurrences) was restricted to central Sweden between 58.35°N and 63.18°N), following a second-order polynomial relationship with latitude (**Figure 1**; **Supplementary Figure S1**; **Table 1C**, question 2).

**Table 1:** GLM with binomial distribution and a log-link function of the effect of latitude (A) on whether the expressed sex is male or female, (B) on sex expression, and (C) on sporophyte occurrences in females (second-order polynomial) in *Abietinella abietina* in Sweden.

Factor	df	Estim (std.err.).	Wald stat.	P	Log-Likelih.	χ^2^	P
A							
Intercept	1	-15.63 (2.47)	40.09	***<0.001***	-245.51		
Latitude	1	0.23 (0.04)	31.66	***<0.001***	-230.00	31.01	***<0.001***
B							
Intercept	1	-1.16 (1.41)	0.68	*0.41*	-689.93		
Latitude	1	0.03 (0.02)	1.31	*0.25*	-689.27	1.32	*0.25*
C							
Intercept	1	-1116.52 (300.16)	13.84	***<0.001***	-166.84		
Latitude	1	36.65 (9.89)	13.72	***<0.001***	-166.77	0.14	*0.75*
Latitude^2^	1	-0.30 (0.08)	13.66	*<* ***0.001***	-149.91	33.72	***<0.001***

Modelled probabilities are expressed sex = male, sex expression = 1, and sporophyte occurrence = 1. Both results of Wald and Log likelihood statistics are presented. A: N = 630 non-sporophytic but sex-expressing samples. B: N = 1032 non-sporophytic samples. C: N = 595 female samples. (Significant) P values in (bold and) italics.

**Figure 2:**
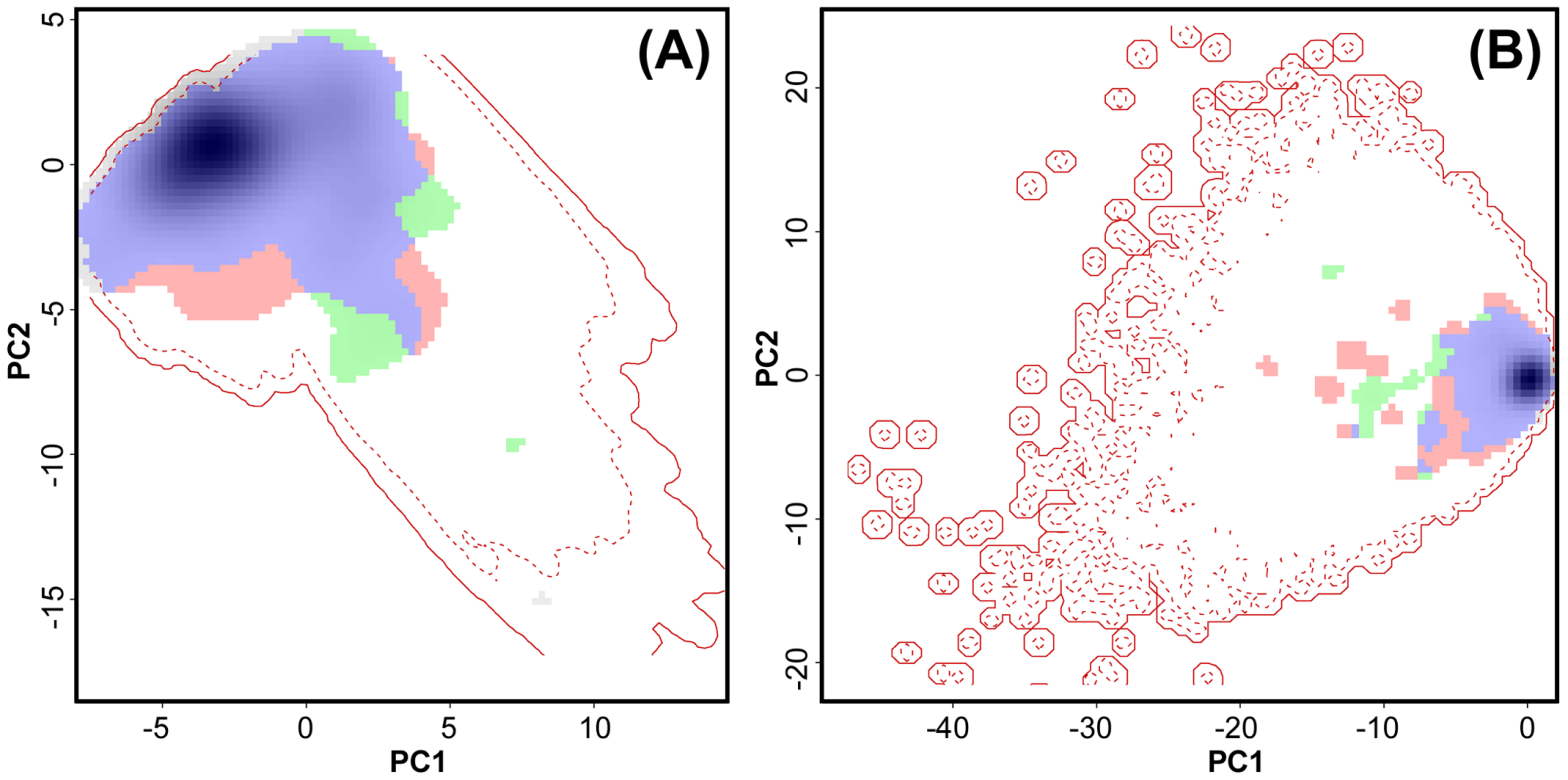
Niche overlap between males and females of *Abietinella abietina* in Sweden. **(A)** Climatic space based on MPI circulation model (see **Supplementary Figure S2** for the UKESM circulation model); **(B)** topographic space. Blue, environmental space in common for males and females (niche overlap); green, part of the male niche not in common with female niche; pink, part of the female niche not in common with the male niche. The degree of shading represents the density of observations. The solid red line corresponds to the extent of the environment conditions in Sweden and the dotted line corresponds to the quantile 75% of the environmental conditions.

However, the different geographic distributions of males and females could not be explained by niche differences (**Figure 2**; question 3). The climatic niche overlaps between males and females were 0.79 and 0.93 according to Schoener’s D and Hellinger’s I metrics, respectively, based on the global circulation model MPI. The corresponding niche overlap metrics were 0.69 (Schoener’s D) and 0.87 (Hellinger’s I) based on the circulation model UKESM. The niche overlaps showed a tendency to be more similar than expected by chance for both global circulation models (MPI: p = 0.052 for Schoener’s D and p = 0.049 for Hellinger’s I; UKESM: p = 0.053 for Schoener’s D and p = 0.041 for Hellinger’s I) (**Figure 2A**; **Supplementary Figure S2**). Schoener’s D and Hellinger’s I were equal to 0.99 and 1, respectively, for the topographic niche overlap (**Figure 2B**). Similarly, the topographic niches were more similar than expected by chance (p = 0.03 for Schoener’s D and p = and 0.02 for Hellinger’s I).

In line with the results for male vs. female occurrences, the topographic and climate niche overlaps between non-expressing and reproductive samples of *A. abietina* were high (Schoener’s D and Hellinger’s I metrics = 0.81 – 0.99) (question 3). The climate niches were more similar than expected by chance (P < 0.001 – 0.02).

Overall sex expression was poorly predicted by climate under both global circulation models (UKESM, MPI) and topography, with maxTSS between 0.19 and 0.23, and AUC between 0.61 and 0.66 (**Supplementary Table S3**). Only RRF models showed strong variation of sex expression along the climatic gradient (**Supplementary Figure S3**): Precipitation seasonality (bio15) and mean monthly precipitation amount (bio19) were the most important predictors with both circulation models (**Supplementary Table S3**). In the GLMs, all predictors had low coefficient values. Precipitation seasonality remained with both MPI and UKESM global circulation models, in addition to annual mean temperature (bio1) in the former and mean monthly precipitation amount (bio19) in the second model (**Supplementary Table S3**).

Contrary to sex expression, the climate and topography models predicted sporophyte occurrences relatively well (model fit metrics: maxTSS >= 0.55; AUC >= 0.82; **Supplementary Table S3**; question 3). The number and order of the variables and their coefficients differed somewhat between the models. Precipitation seasonality (bio15) and the cosine of aspect, reflecting the South-North axis, remained in all four models (**Supplementary Table S3**). Of these, bio 15 was most or second most highly ranked in both RRF models. In the model based on UKESM, minimum temperature in the coldest month (bio6) was selected with highest rank. Sporophyte occurrence probability was lower at both lowest and highest precipitation seasonality and temperature values in RFF (**Supplementary Figure S4**). In the GLM, temperature values were the most important predictors for sporophyte occurrence and followed similar trends as temperature values in RFF, while sporophyte occurrence was positively correlated to precipitation seasonality. In the GLM, (**Supplementary Table S3**), intermediate values of the minimum temperature in the coldest month (bio6) and of annual mean temperature (bio 1) predicted sporophyte formation best in the GLM based on the UKESM, and in the GLM based on the MPI circulation model, respectively (**Supplementary Figure S4**).

## Discussion

More than 60% of our samples, representing the Swedish population of *A. abietina*, formed sexual organs, phenotypically female samples outnumbered male samples by a factor 5.6 and sporophytes were rare. While overall sex expression was not associated with latitude, the probability of being male among the reproductive samples increased with latitude, resulting in a higher regional sex ratio in the North compared to southern regions. However, the distribution of the sexes was not explained by topographic or climate niches modelled here. Successful sexual reproduction, indicated by the occurrence of sporophytes, was confined to central Sweden and was predicted by intermediate to increasing precipitation seasonality and intermediate temperature values.

### Sex expression, the distribution of phenotypic females and males and sex ratios

The sex expression level of 63% in *A. abietina* is at the highest end compared to other unisexual long-lived mosses, and higher than we expected given the low sporophyte formation (During, 1979; Bisang and Hedenäs, 2005; Bisang et al., 2014). Considering only the area of sporophyte production in central Sweden (**Supplementary Figure S1**), the proportion of sporophytic samples among females and overall sex expression were 11% and 66%, respectively. The latter is higher than in north (58%) and south Sweden (56%). This indicates that increased sex expression, i.e., functional mate availability, enhances fertilization incidence in *A. abietina*, as shown in a previous experimental field study (Bisang et al., 2004), and for other species (Cronberg, 2002; Bisang and Hedenäs, 2005; Rosengren and Cronberg, 2014; Bisang et al., 2020). The map in **Figure 1** displays a stronger overlap of the sexes in central than in northern and southern Sweden. Fertilization rate is expected to depend on the relative numbers and distribution of male and female mates. However, few other studies have rigorously compared the relationship between sex ratios and sporophyte frequencies in large bryophytes samples, likely since the inconspicuous pre-zygotic reproductive structures are laborious to assess. Within-species variation in sex ratio was associated with sporophyte frequency in a liverwort (Blackstock, 2015) and a moss (Boquete et al., 2021), both long-lived species. No such relationship was found in a limited one-population study of a dryland moss (dos Santos et al., 2022). This insinuates that reproductive performance is species-specific and, as discussed below, shaped by additional external factors (e.g., Yang and Kim, 2016; Wyatt et al., 2023).

The strong female bias in sex-expression agrees with our prediction and previous evidence in most unisexual bryophytes (Bisang and Hedenäs, 2005; Glime and Bisang, 2017; de Jong et al., 2018). Different causes have been proposed to explain the common male rarity in bryophyte populations (Bisang et al., 2014; for a summary). During the past decades, the postulation that male bryophytes have a lower survival rate, especially in ‘extreme’ environments, due to a higher pre-zygotic investment in sexual reproduction than females, gained recurrent attention (termed ‘cost of realized sexual reproduction’-hypothesis: Stark et al., 2000). This was based on studies of sex expression in the desert moss *Syntrichia caninervis* Mitt. In *A. abietina*, among the samples forming reproductive organs, the probability of being male increases, and of being female decreases northwards, resulting in geographic sex ratio variation. The northern part of our study region in Sweden extends to 68.52°N in the cold-temperate zone. It may be considered more extreme compared to the warm-temperate (nemoral) zone further south, for example in terms of growing season, low temperatures or temperature fluctuations (Dierssen, 1996). In several other bryophyte species, male plants were more common in unfavorable sites than females (Boquete et al., 2016; Bisang et al., 2020; Boquete et al., 2023) or less sensitive to adverse experimental conditions (Cameron and Wyatt, 1990; Stark et al., 2009). Patterns of population sex ratio variation along ecological gradients has also been observed in dioecious and sexually polymorphic flowering plants, where sex-specific selection has been proposed as the driving force (e.g., Caruso and Case, 2007; Yakimowski and Barrett, 2014). Such findings suggest that sex-specific selection could similarly shape bryophyte populations, particularly in environments where the resource demands of female plants, due to their role in nurturing sporophyte offspring, are higher (Ehrlén et al., 2000; Rydgren and Økland, 2002a; Barrett and Hough, 2013).

If we assume that male plants are not truly rare in *A. abietina*, but rather form sexual organs much more scarcely than females (‘‘shy male hypothesis’’; Bisang and Hedenäs, 2013), we can assign male sex to all non-expressing samples, resulting in a balanced sex ratio (0.48). This would not violate the expected even sex distribution at germination (Bachtrog et al., 2011; Bisang et al., 2017) but differs from recent findings that suggest that genotypic and phenotypic sex ratios do not differ in adult perennial bryophytes (e.g., Bisang et al., 2023; Boquete et al., 2023). Molecular methods that allow to distinguish between effects on sex expression (sex phenotypes) and on sex genotypes (Norrell et al., 2014; Bisang et al., 2020; Ekwealor et al., 2022) have not yet been developed for *A. abietina*. This, and including a wide array of species with different life histories and from various environments as outlined above, will lead to a more accurate picture of bryophyte sex ratio variation and its multiple drivers (Baughman et al., 2017; Bisang et al., 2020; Boquete et al., 2023; Dorken, 2023).

### Topographic and climate niches and effects of topo-climate factors on reproductive stages

Contrary to our expectation, and despite the clear latitudinal pattern in male sex expression, the overlap of the climatic and topographic niches between males and female *A. abietina* was high in all models based on different global circulation models, modelling approaches and model fit metrics. The same held true for samples with and without reproductive organs (sex expression). In line with our findings, the sexes and reproductive modes in the perennial moss *Pseudocsleropodium purum* (Hedw.) M. Fleisch. showed poor niche differentiation (Boquete et al., 2023). It suggests that other than these macroscale environmental conditions account for the differences in male and female distributions. Factors at smaller scales interact with macroenvironmental conditions and shape local microclimates (Kemppinen et al., 2024, for an overview). Evidence increasingly reveals large differences between macroclimate and the conditions that organisms actually experience (microclimate), due to local variation in topography, substrate, wind, light or vegetation structure (Lembrechts, 2023; Collart et al., 2024). Such factors can have a more pronounced effect on the distribution and development of organisms than macroclimate, including *A. abietina* and other bryophytes that often depend on microhabitat conditions (Greiser et al., 2020; Haesen et al., 2023; Collart et al., 2024). Reproductive performance in bryophytes has been shown to be affected by chemical factors in the substrate and by various physical factors (Chopra and Bhatla, 1981; 1983; Sundberg, 2002). Light conditions and photoperiod were demonstrated to affect the initiation and formation of gametangia (Chopra and Bhatla, 1981; Hohe et al., 2002; Rydgren and Økland, 2002b). Because male *A. abietina* plants occur across almost the entire latitudinal gradient, photoperiod cannot be the single trigger to induce male gametangia. In line with this, sex-specific immigration routes seem unlikely given the wide distribution of both sexes (Bisang et al., 2023; Boquete et al., 2023; Collart et al., 2024). However, historical and light or radiation factors, combined with other environmental variation, e.g., snow cover, microtopography, substrate, distance from the sea, vegetation or other biotic parameters, which we did not catch with the present models, are likely to contribute to the relatively greater male expression frequency in higher latitudes.

### Successful sexual reproduction

Our results indicated that, beyond mate availability, topo-climate factors were critical for fertilization and sporophyte maturation to occur. This aligns with our expectation based on extensive field observations, and published results from different plant groups (Elmqvist et al., 1988; Caruso and Case, 2007; Yakimowski and Barrett, 2014; Pereira et al., 2016; Bisang et al., 2020). For example, successful sexual reproduction is often lower, in favor of clonality, towards range margins where species may experience different or more stressful environments than in their distribution center (Silvertown, 2008; Yakimowski and Barrett, 2014). In the monoicous *Pohlia nutan*s (Hedw.) Lindb., a species with a wide ecological amplitude, sporophyte production decreased the closer to geothermal features the species occurred (Eppley et al., 2011). The authors suggested that the physiological stress response related to the distance to the stressor interacted with species identity and sex expression level. In the long-lived unisexual tropical *Syrrhopodon involutus* Schwägr., however, sporophytes were as frequent at the margin compared to the center of its range, but female sex expression declined towards the margin (Fisher, 2011). In this case, mate availability seemed more important for reproductive success than environmental conditions. In our study species, the regionally abundant sporophyte occurrence in an East-West belt in central Sweden that extends westwards to Norway (Gudbrandsdalen: https://artsdatabanken.no/Pages/305542/Abietinella_abietinum, accessed 2024-06-08; pers. observ. LH, IB) is intriguing. The species is widespread and common on base-rich substrates across the Holarctic, while sporophytes are consistently reported to be rare or extremely rare (Buck, 2014; Hedenäs et al., 2014; Ignatov et al., 2022; Limpricht, 1890-1895). Important predictors in the topo-climate models relate to moisture and temperature conditions. *Abietinella abietina*, as most bryophytes, lacks an internal conductive system and depends on water in its environment to transport water and assimilates (poikilohydric), and sperms are passively transported in a water film or capillary system of paraphyllia and leaves to reach the egg (Bisang et al., 2004; Vanderpoorten and Goffinet, 2009; Glime and Bisang, 2017; Brodribb et al., 2020). Thus, adequate humidity levels and timing and duration of humid periods are required for fertilization to happen, the sporophyte to develop (Sundberg, 2002; Hedenäs and Bisang, 2019; Bisang and Hedenäs, 2022) and the plants to accumulate resources for growth and survival (Proctor, 2009). Plant size, affected by water availability, likely affects both sex expression and sporophyte formation, through resource supply and threshold size for sexual reproduction (Bisang and Ehrlén, 2002; Rydgren and Økland, 2002b; Boquete et al., 2023). Like many other bryophytes, *A. abietina* is cold-resistant and desiccation-tolerant, indicating that temperature effects on sporophyte formation were indirect through influence on moisture conditions (Proctor, 2009; Stark, 2017). Although the model fits to predict sex expression by climatic and topographic factors were quite low in this study, precipitation seasonality remained as predictor with a relatively high coefficient in all models, plus monthly precipitation amount. This was not unexpected in light of the reproductive biology of *A. abietina*, and was reported for other unisexual bryophytes, e.g., six species of Calymperaceae in lowland Amazonia (Pereira et al., 2016). For our focus species *A. abietina*, we can currently not assess the relative contributions of functional mate deficiency, environmental conditions or other factors to the lack of successful sexual reproduction in most of the species’ range, as pre-zygotic sex expression was not assessed in other areas than that covered by this study.

One could argue that investment in pre-zygotic reproductive structures was a waste of resources if conditions for the accomplishment of successful sexual reproduction seem rare. It was suggested that genetic sterility evolves following situations when sexual recruitment is inhibited by ecological conditions (e.g., Eckert, 2002). However, genetic infertility may be transient or incomplete and interact with environmental factors (Dorken and Eckert, 2001; Charpentier, 2002; Eckert, 2002; Barrett, 2015). In *A. abietina*, the widespread formation of pre-zygotic sexual organs could be considered a type of reproductive assurance. Since the demographic cost of their formation is limited compared to sporophyte production (Rydgren and Økland, 2002a; 2003; Bisang et al., 2006; dos Santos et al., 2022), they could be produced, and the plants remain ready for realizing fertilization if conditions allow. Contrary to most seeds, spores are easily wind-dispersed over long distances, implying that even occasional sexual reproduction contributes to maintain relevant levels of genetic diversity across species’ ranges (Hedenäs and Bisang, 2019; Fichant et al., 2023). Thus, even if successful sexual reproduction is spatially limited in bryophytes, this likely entails less strict constraints on genetic structuring and resulting reduced fitness than in seed plants (Eckert, 2002; Silvertown, 2008; Patiño and Vanderpoorten, 2018; Vanderpoorten et al., 2019).

## Conclusions and perspectives

In the long-lived unisexual moss *A. abietina*, representing the only land plant lineage that manifests dioicy in the dominant haploid gametophyte generation, female and male sexual niches were not differentiated. Yet, the probability of being male among sex expressing plants increased and of being female decreased with increasing latitude. It implies that the macroscale topo-climate factors included in the models here did not account for these sex-specific distributions. Pre-zygotic sex expression did not exhibit a spatial pattern. Sporophyte occurrences, resulting from successful sexual reproduction, were not only rare overall, but also spatially confined to the central part of Sweden, along the latitudinal gradient from warm- to cold-temperate climate zones. They depended on female and male mate availability and macroenvironmental factors related to moisture conditions and slope orientation along the South-North axis. The species thus appears to spread mainly clonally in most parts of its distribution range. Since moss spores are wind dispersed, occasional sexual recruitment may occur even in areas without spore production. This could counteract negative effects due to ‘failure of sexual reproduction’ observed in largely clonally reproducing seed plants, especially in unisexual species exhibiting sporophytic dioecy (Barrett, 2015). We hypothesize that the distribution of reproductive stages in this moss is governed by an interaction of environmental factors at both macro- and smaller scales with biological attributes, such as that fertilization depends on sex expression, and that sporophyte production may trade-off against future performance. We suggest that the uneven distribution of the reproductive stages has lower than expected effects on the species long-range dispersal and overall distribution in this occasionally wind-dispersed plant. We plan to explore these postulations by i) modelling the distribution of reproductive stages using environmental data at finer resolution and additional parameters (e.g., light, substrate); ii) extending the study region to include the sporophyte-rich area in west-neighboring Norway; and iii) comparing genetic diversity in sporophytic with non-sporophytic populations.

## Data Availability

The original contributions presented in the study are included in the article/**Supplementary Material**. Further inquiries can be directed to the corresponding author.
