# Supplementary material for: Factors accounting for limited sexual reproduction in a long-lived unisexual plant species
Source: Front Plant Sci. 2025 Feb 11;16:1456877. doi: 10.3389/fpls.2025.1456877 (PMC11850334; doi:10.3389/fpls.2025.1456877)
Supplement: Supplementary file 1 [file DataSheet1.pdf]

Supplementary Material

Bisang, Collart, Vanderpoorten & Hedenäs:

Factors accounting for limited sexual reproduction in a long-lived unisexual plant species

Table S2

Table S3

Figure S1

Figure S2

Figure S3

Figure S4

Table S2

Climatic (C) and topographic (T) variables used in the niche modelling
https://luizfesser.wordpress.com/2021/03/08/the-19-bioclimatic-variables/

		Variable	Unit	Description
**C**	bio1	Annual mean air temperature	°C	Mean annual daily mean temperatures over 1 year; averaged across 20-year period
**C**	bio2	Mean diurnal range of temperatures	°C	Mean diurnal range of temperatures over 1 year, i.e., mean of monthly (max temp – min temp); averaged across 20-year period
**C**	bio3	Isothermality	°C	Ratio of diurnal variation to annual variation in temperatures over 1 year; averaged across 20-year period
**C**	bio4	Temperature seasonality	°C/100	Standard deviation of the monthly mean temperatures over 1 year; averaged across 20-year period
**C**	bio5	Maximum temperature of warmest month	°C	Maximum temperature over 1 year; averaged across 20-year period
**C**	bio6	Minimum temperature of coldest month	°C	Minimum temperature over 1 year; averaged across 20-year period
**C**	bio7	Annual range of temperature	°C	Difference between the Maximum temperature of warmest month and the Minimum temperature of coldest month over 1 year; averaged across 20-year period
**C**	bio8	Mean temperature of the wettest quarter	°C	The wettest quarter of the year determined over 1 year; averaged across 20-year period

**C**	bio9	Mean temperature of the driest quarter	°C	The driest quarter of the year determined over 1 year; averaged across 20-year period
**C**	bio10	Mean temperature of the warmest quarter	°C	The warmest quarter of the year determined over 1 year; averaged across 20-year period
**C**	bio11	Mean temperature of the coldest quarter	°C	The coldest quarter of the year determined over 1 year; averaged across 20-year period
**C**	bio12	Annual precipitation	kg m^{-2}	Sum of monthly precipitation amounts over 1 year; averaged across 20-year period
**C**	bio13	Precipitation amount of the wettest month	kg m^{-2}	The precipitation amount of the wettest month over 1 year; averaged across 20-year period
**C**	bio14	Precipitation amount of the driest month	kg m^{-2}	The precipitation amount of the driest month over 1 year; averaged across 20-year period
**C**	bio15	Precipitation seasonality	kg m^{-2}	The coefficient of variation of the monthly precipitation amounts over 1 year; averaged across 20-year period
**C**	bio16	Precipitation amount of the wettest quarter	kg m^{-2}	The wettest quarter of the year is determined over 1 year; averaged across 20-year period
**C**	bio17	Precipitation amount of the driest quarter	kg m^{-2}	The driest quarter of the year is determined over 1 year; averaged across 20-year period
**C**	bio18	Precipitation amount of the warmest quarter	kg m^{-2}	The warmest quarter of the year is determined over 1 year; averaged across 20-year period
**C**	bio19	Precipitation amount of the coldest quarter	kg m-2	The coldest quarter of the year is determined over 1 year; averaged across 20-year period
**T**		Aspect cosine		Cosine of the aspect, reflecting the axe South-North
**T**		Aspect sine		Sine of the aspect, reflecting the axe West-East.
**T**		Elevation	m [asl]	
**T**		Slope	Degree	
**T**		Northness		Cosine of aspect * sine of the slope; Comprised between -1 (South face with a strong slope) and 1 (North face with a strong slope)

**T**		Eastness		Sine of aspect * sine of slope; Comprised between -1 (West face with a strong slope) and 1 (East face with a strong slope)
**T**		Profile Curvature		Reflects the concavity and convexity in the direction of the slope
**T**		Tangential Curvature		Reflects the concavity and convexity in the direction perpendicular to the slope
**T**		Topographic Position Index	m	Difference between the elevation of a focal cell and the mean of its 8 surrounding cells. Positive and negative values correspond to ridges and valleys, respectively
**T**		Terrain Ruggedness Index	m	mean of the absolute differences in elevation between a focal cell and its 8 surrounding cells
**T**		Roughness	m	Largest inter-cell difference of a focal cell and its 8 surrounding cells
**T**		Vector Ruggedness Measure		Quantifies terrain ruggedness by measuring the dispersion of vectors orthogonal to the terrain surface.

Top

Table S3

Model metrics of Generalized Linear Models (GLM) and Regularized Random Forest Models (RRF) to predict the presence of sex expression and the occurrence of sporophytes, indicating successful sexual reproduction, in *Abietinella abietinum* in Sweden based on topographic and climate parameters using two circulation models (MPI, UKESM). Colour coding depicts predictors that were selected as important in several models.

Response variable	Model	Circulation models	Evaluation of the fit	Coefficient GLM/ mean decrease in Gini coefficient RRF
Sex expression	GLM	MPI	AUC = 0.62<br>maxTSS = 0.19	Bio1 = 1.96<br>*Bio15 = 1.87*
		UKESM	AUC = 0.61<br>maxTSS = 0.19	*Bio15 = 4.74*<br>Bio19 = 1.76
	RRF	MPI	AUC = 0.66<br>maxTSS = 0.23	*bio15 = 56.86*<br>bio19 = 47.75<br>aspectsine = 44.03<br>roughness = 42.33<br>northness = 40.58<br>bio9 = 39.84<br>pcurv = 39.35<br>bio7 = 38.5<br>bio1 = 37.88<br>aspectcosine = 35.48<br>bio8 = 32.96<br>bio5 = 31.43
		UKESM	AUC = 0.64<br>maxTSS = 0.21	Bio19 = 52.28<br>*Bio15 = 50.79*<br>Roughness = 43.52<br>Aspectsine = 41.98<br>Northness = 40.94<br>Pcurv = 39.80<br>Bio9 = 39.78<br>Bio3 = 36.33<br>Aspectcosine = 35.45<br>Elevation = 35.13<br>Bio2 = 32.61
Sporophyte occurrence (successful reproduction)	GLM	MPI	AUC = 0.87<br>maxTSS = 0.62	bio1 = 8.19<br>aspectcosine = 7,32<br>bio9 = 6,04<br>*bio15 = 4,90*<br>bio8 = 2,39<br>bio14 = 2,11<br>tpi = 1,62<br>tcurv = 0,98
		UKESM	AUC = 0.82	bio6 = 7,81

			maxTSS = 0.55	bio8 = 4,92<br>aspectcosine = 4,78<br>*bio15 = 4,41*<br>bio5 = 3,07<br>tpi = 0,94<br>tcurv = 0,22
	RRF	MPI	AUC = 0.85<br>maxTSS = 0.57	*Bio15 = 27.37*<br>Bio9 = 23.33<br>Tpi = 13.26<br>Aspectcosine = 10.93
		UKESM	AUC = 0.84<br>maxTSS = 0.58	Bio6 = 21.23<br>*Bio15 = 19.70*<br>Aspectcosine = 7.54<br>Northness = 7.13

Top

Figure S1

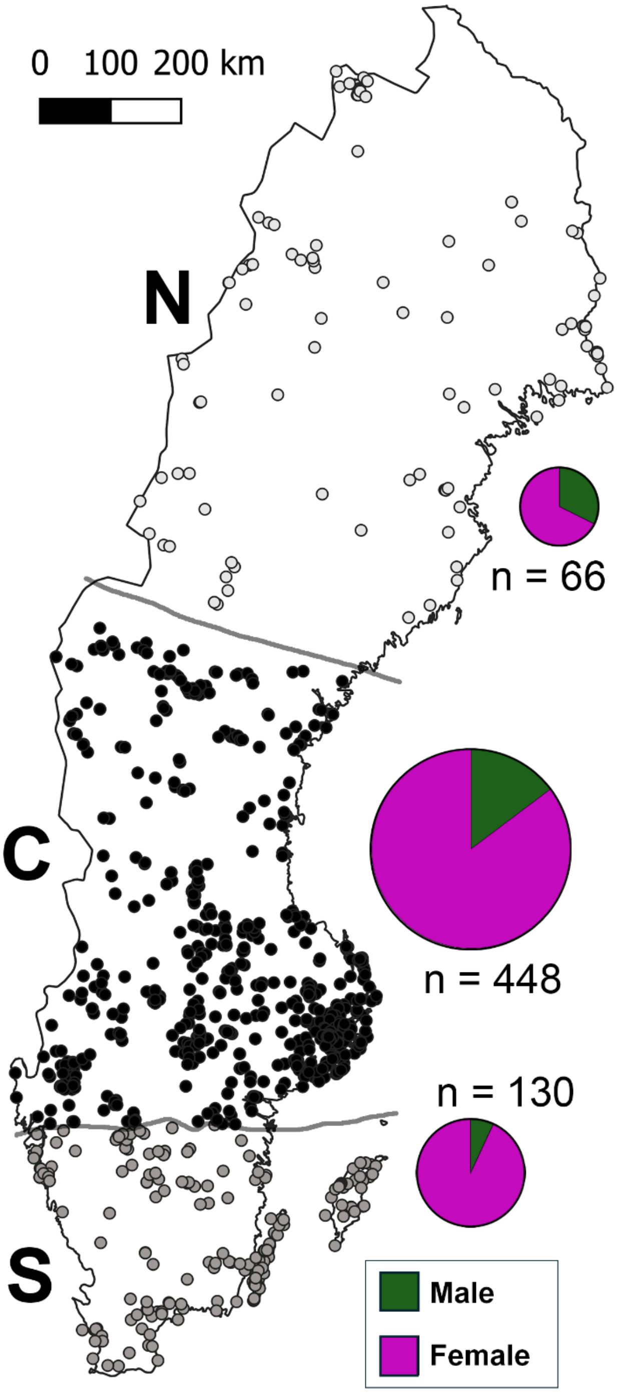

Distribution of 1,130 sampling localities of *Abietinella abietina* in three regions in Sweden (S, southern; C, central; N, northern Sweden). The pie diagrams, with a diameter relative to the number of sex expressing samples, depict sex expressing male (green) and sex expressing female (pink) samples. They indicate variation in sex expression levels (S, 56%; C, 66%; N, 58%) and that sex ratio is increasing from south to north Sweden due to a relatively higher proportion of male samples.

Top

Figure S2

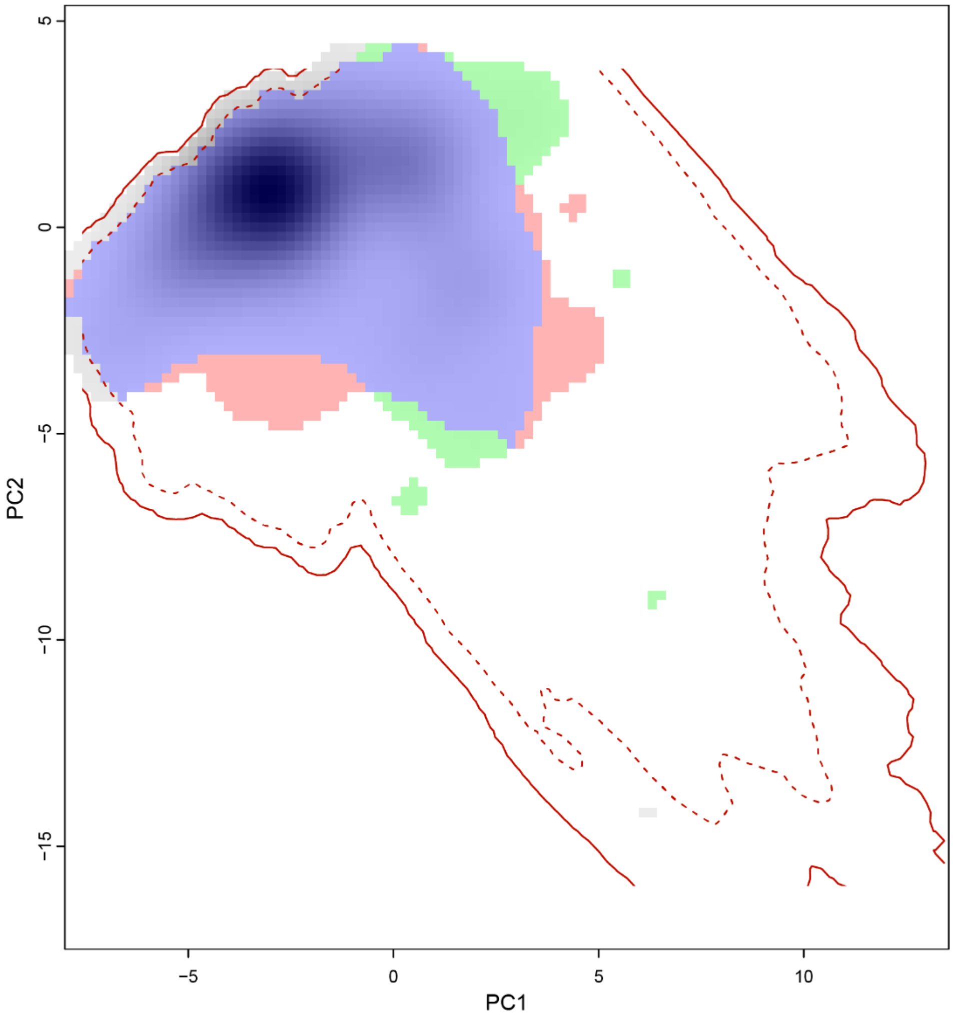

Climate niche overlap between males and females of *Abietinella abietina* in Sweden. Climatic space based on UKSEM circulation model. Blue, environmental space in common for males and females (niche overlap); green, part of the male niche not in common with female niche; pink, part of the female niche not in common with the male niche. The degree of shading represents the density of observations. The solid red line corresponds to the extent of the environment conditions in Sweden and the dotted line corresponds to the quantile 75% of the environmental conditions.

Top

Figure S3

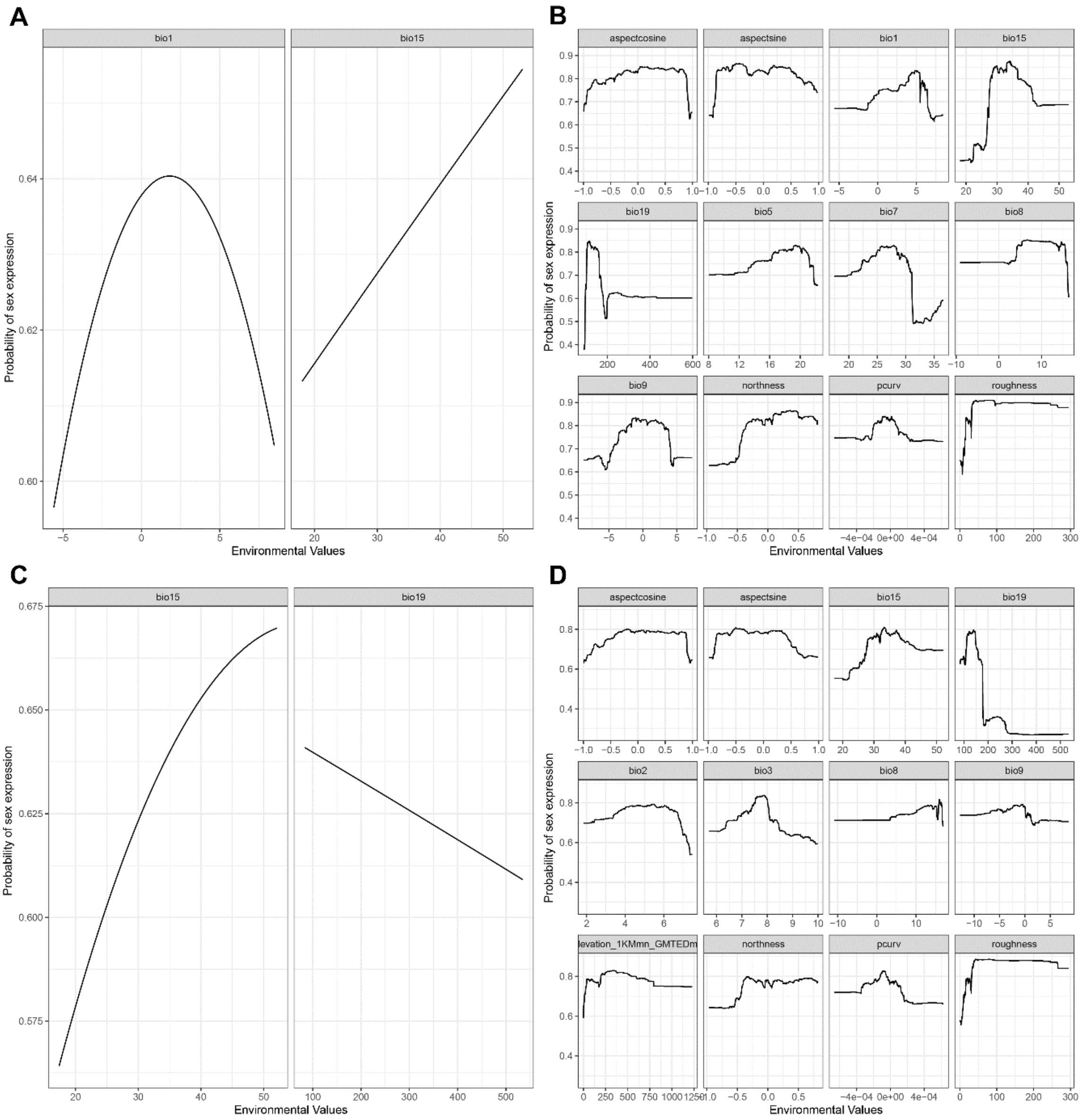

Response curves for each predictor used to explain **sex expression** using (A, C), GLM, or (B, D), Regularized Random Forest models. Circulation models: (A, B), MPI; (C, D) UKESM.

Topo-climate predictors (alphabetically; Table S2 for further explanations): Aspectcosine [aspectsine], cosine [sine]of the aspect, reflecting the axis South-North [West-East]; bio1, annual mean temperature; bio2, mean diurnal temperature range; bio3, isothermality; bio5, maximum air temperature of the warmest month; bio7, annual range of air temperature; bio8[bio9], mean temperature of the wettest [driest]quarter; bio15, precipitation seasonality; bio19, mean monthly precipitation amount of the coldest quarter; elevation, masl; northness, cosine of aspect * sine of the slope; pcurv, profile curvature; roughness, largest intercell difference of a focal cell and surrounding cells. Top

Figure S4

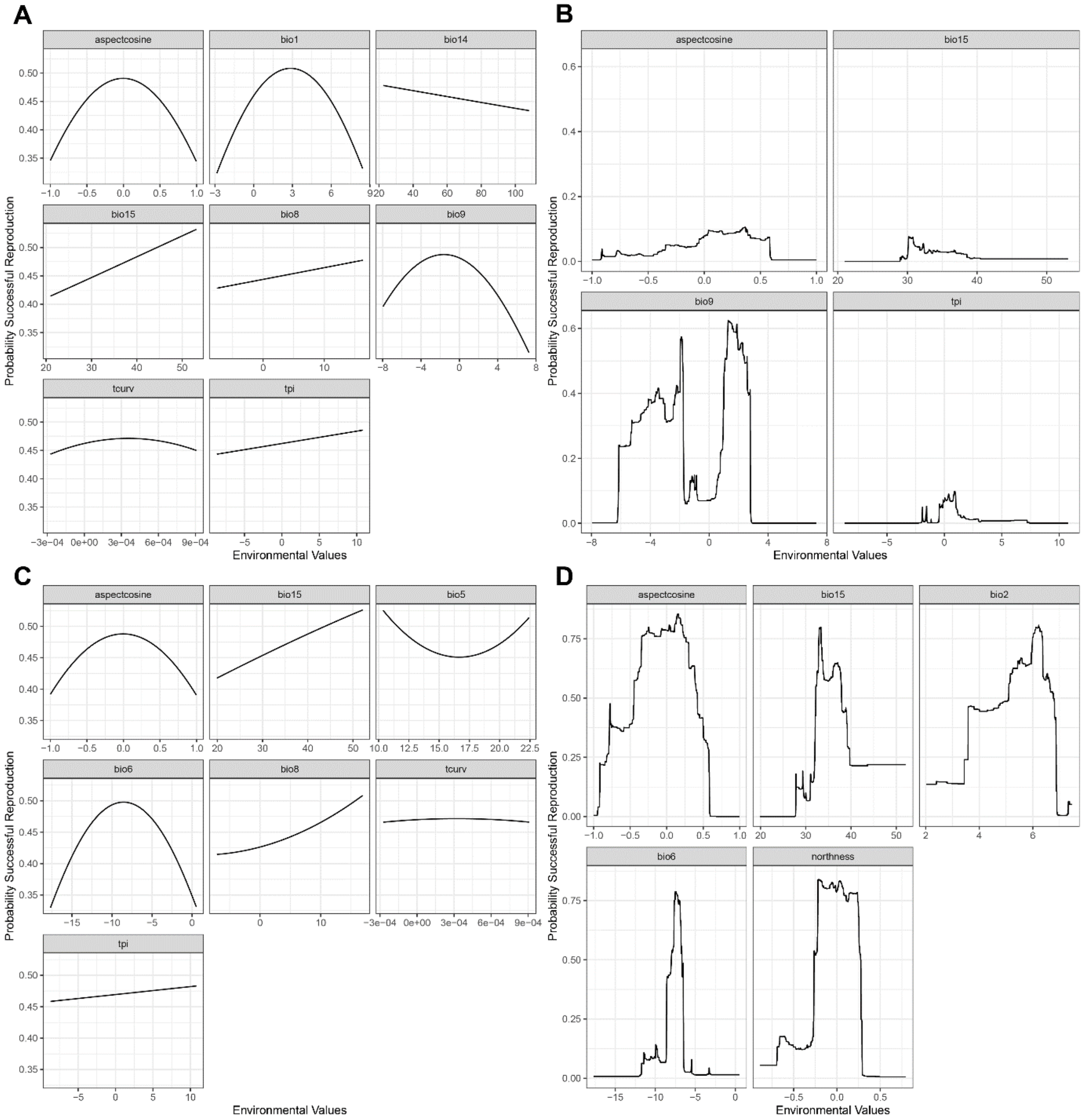

Response curves for each predictor used to explain **successful sexual reproduction (sporophyte occurrences)** using (A, C), GLM, or (B, D), Regularized Random Forest models. Circulation models: (A, B), MPI; (C, D) UKESM.

Topo-climate predictors (alphabetically; Table S2 for further explanations): *Aspectcosine,* cosine of the aspect, reflecting the axe South-North; *bio1*, annual mean temperature; *bio2,* mean diurnal temperature range; *bio5,* maximum air temperature of the warmest month; *bio6,* minimum temperature of the coldest month; *bio8* [*bio9*], mean temperature of the wettest [driest] quarter; *bio14*, precipitation amount of the driest month; *bio15*, precipitation seasonality; *northness*, cosine of aspect * sine of the slope; *tcurv*, tangential

curvature; reflecting concavity and convexity perpendicular to the slope; *tpi*, Topographic Position Index, i.e. the elevation difference between the focal cell and the mean of eight surrounding cells.Top
